# Commentary: Lactylation-related gene *AKR1A1* contributes to osteoporosis via metabolic–immune regulation: evidence from multi-omics integration, single-cell transcriptomics, and *in vitro* validation

**DOI:** 10.3389/fimmu.2025.1752400

**Published:** 2026-01-16

**Authors:** Jintao Cui, Bin Xu, Penghui Zhang, Songfu Han, Chaoyanng Guo, Limin Wei

**Affiliations:** Department of Oncology, The First Affiliated Hospital of Henan University of Science and Technology, Luoyang, China

**Keywords:** AKR1A1, lactylation (Kla), osteoporosis, post-translational modification, single cell

Osteoporosis is characterized by complex metabolic reprogramming and dysregulation of the immune microenvironment; however, its molecular mechanisms remain incompletely understood, highlighting the need for novel diagnostic biomarkers and therapeutic targets. Recently, Shao et al. integrated bulk transcriptomic and single-cell RNA sequencing data to identify differentially expressed lactylation-related genes and constructed a diagnostic model using multiple machine-learning approaches, proposing *AKR1A1* as a potential key feature gene in osteoporosis. By combining single-cell clustering, CellChat-based cell–cell communication analysis, and *in vitro* experiments using RAW264.7 macrophages, the authors suggest that *AKR1A1* lactylation may be involved in osteoporosis-related processes through the SPP1–CD44 signaling axis and broader metabolic–immune networks (1). While this study provides exploratory insights from the perspective of protein lactylation, several aspects warrant further discussion, particularly with regard to ethical reporting, mechanistic validation, and the representativeness of the single-cell data.

First, in the *in vitro* experiments, RAW264.7 cells were treated with serum from patients with osteoporosis to mimic a disease-associated microenvironment and to assess its effects on *AKR1A1* expression and lactylation. While this design is clinically relevant, the manuscript provides limited information regarding the human serum samples. The Methods section does not clearly specify sample sources, participant recruitment and exclusion criteria, serum processing procedures, or details of ethical approval and informed consent. According to internationally recognized guidelines, including the Declaration of Helsinki and the STROBE statement, studies involving human biological materials should explicitly report sample provenance and ethical compliance (2, 3). To improve transparency and reproducibility, future work should clarify donor demographics (e.g., age, sex, and menopausal status), diagnostic criteria for osteoporosis (such as DXA T-scores ≤ − 2.5 and the exclusion of secondary osteoporosis), key disease characteristics, relevant comorbidities and medications affecting bone metabolism, as well as the approving ethics committee, approval number, and informed consent procedures.

Second, previous studies have implicated *AKR1A1* in bone metabolic homeostasis, including its roles in osteogenic–adipogenic differentiation, oxidative stress regulation, and vitamin C-related metabolism, providing functional context for its relevance in bone biology (4–6). However, alterations in protein abundance alone do not directly establish the functional significance of a specific posttranslational modification, which is typically dynamic and site dependent. In the study by Shao et al., *AKR1A1* was identified as a key feature gene, but experimental validation was largely limited to expression analysis and pathway inference. The absence of *AKR1A1*-specific interventional experiments, such as knockdown, overexpression, or site-directed mutagenesis of lactylation sites, makes it difficult to disentangle the respective contributions of protein abundance and lactylation modification (7). Previous studies on protein lactylation have emphasized the importance of functional perturbation strategies to establish causality (8). Accordingly, future studies incorporating lactylation-deficient mutants or rescue experiments would be critical for defining the mechanistic role of *AKR1A1* lactylation in metabolic–immune regulation.

Third, the authors analyzed a publicly available single-cell RNA sequencing (scRNA-seq) dataset (GSE147287) derived from a single sample to assess cell-type–specific *AKR1A1* expression and infer cell–cell communication networks. Although scRNA-seq is well suited for characterizing cellular heterogeneity, analyses based on a single donor are limited in capturing interindividual variability and may be influenced by sample-specific factors (9). Consequently, inferred interactions such as the SPP1–CD44 signaling axis may have restricted robustness and generalizability. Integrating scRNA-seq data from multiple donors with diverse clinical and demographic characteristics would enable more reliable validation of *AKR1A1*-associated cellular interactions.

Overall, Shao et al. present exploratory evidence linking protein lactylation to metabolic–immune regulation in osteoporosis and propose *AKR1A1* as a gene of potential interest based on multi-omics and single-cell analyses. While the study offers a novel conceptual framework, limitations in ethical reporting, mechanistic validation, and data representativeness remain. Addressing these issues through comprehensive ethical documentation, targeted functional experiments focusing on *AKR1A1* lactylation, and validation across multiple scRNA-seq datasets will be essential to more rigorously assess the role of *AKR1A1* lactylation in osteoporosis and its potential translational relevance.
